# Exosomal transfer of macrophage-derived NEAT1 enhances DNA damage response and confers cisplatin resistance in lung adenocarcinoma via the MAD1L1/p53 axis

**DOI:** 10.7150/ijbs.128214

**Published:** 2026-03-25

**Authors:** Yi Yang, Min Meng, Yi Zhao, Fangyuan Yu, Harsh Patel, Zhe-Sheng Chen

**Affiliations:** 1 Shanghai Lung Cancer Center, Shanghai Chest Hospital, Shanghai Jiao Tong University School of Medicine, Shanghai, China.; 2 Department of Oncology, Shandong Provincial Hospital Affiliated to Shandong First Medical University, Jinan, Shandong, China.; 3 Department of Pharmaceutical Sciences, College of Pharmacy and Health Sciences, St. John's University, Queens, New York, USA.

**Keywords:** NEAT1, macrophages, exosomes, chemotherapy resistance, DNA damage repair, apoptosis

## Abstract

Cisplatin (DDP) resistance remains a major therapeutic obstacle in non-small-cell lung cancer (NSCLC). Tumor-associated macrophages (TAMs) are known to promote chemoresistance via exosomal signals, but whether exosomal long non-coding RNA NEAT1 contributes to this process is unclear. In this study, we found that exosomes derived from DDP-treated macrophages were enriched with NEAT1 and delivered it to A549 cells. This transfer enhanced the DNA damage response, promoted cell-cycle progression, and reduced DDP-induced apoptosis. Through RNA-sequencing and luciferase reporter assays, we identified MAD1L1 as a key downstream target of NEAT1. NEAT1 was enriched at the MAD1L1 promoter, upregulated its expression, and subsequently suppressed the p53/p21/Bax axis, thereby fostering a chemoresistant phenotype. *In vivo*, exosomal NEAT1 promoted tumor growth in DDP-treated xenografts, while NEAT1 knockdown reversed this effect and restored p53 pathway activity. Collectively, our work unveils a novel TAM-exosome-NEAT1-MAD1L1/p53 signaling axis that drives cisplatin resistance in lung adenocarcinoma, highlighting NEAT1 and its intercellular delivery as potential therapeutic targets to overcome chemoresistance.

## Introduction

Lung cancer remains the most prevalent malignancy worldwide and is the leading cause of cancer-related mortality^1^-^3^. Owing to its insidious onset and lack of symptoms at early stages, most patients are diagnosed with advanced non-small-cell lung cancer (NSCLC)^4^-^6^. By this time, surgical resection is no longer a viable option. For these patients, cisplatin (DDP)-based chemotherapy remains a mainstay and is often administered in combination with other cytotoxic agents^7^-^9^. However, approximately 10-20% of patients exhibit primary resistance to DDP, and an additional 30-50% develop acquired resistance during treatment^10^. This severely limits long-term therapeutic efficacy and highlights the need to better understand resistance mechanisms^11^. Traditional models of DDP resistance include upregulation of multidrug resistance proteins, enhanced DNA damage repair^12^, ^13^, and tumor heterogeneity^14^. More recently, attention has shifted toward the tumor microenvironment (TME), which plays a central role in modulating drug response and tumor progression^8^, ^15^. Among the cellular constituents of the TME, tumor-associated macrophages (TAMs) are particularly abundant in NSCLC and have emerged as key regulators of chemoresistance. Through the secretion of cytokines and extracellular vesicles (EVs), including exosomes, TAMs actively remodel the tumor microenvironment (TME) and influence cancer cell behavior^16^, ^17^.

Growing evidence suggests that TAM-derived exosomes can modulate tumor invasion, metastasis, and drug sensitivity^18^, ^19^. However, most previous studies have focused on exosomes from undisturbed macrophages without examining how chemotherapeutic stress alters the composition or function of these vesicles. Notably, exosomes from drug-stimulated TAMs may act as potent conveyors of adaptive resistance. Therefore, clarifying the role of DDP-induced TAM exosomes and their molecular cargo is crucial for understanding therapy resistance in lung cancer^20^-^22^.

Long non-coding RNAs (lncRNAs) are emerging as major effectors in intercellular communication^23^, especially via exosomal transfer^24^. These RNAs regulate gene expression through diverse mechanisms^25^, including chromatin remodelling, transcriptional control, and protein scaffolding. Among them, nuclear enriched abundant transcript 1 (NEAT1) is highly expressed in NSCLC tissues and correlates with poor prognosis^26^, ^27^. NEAT1 has also been shown to mediate chemoresistance in NSCLC by activating the AKT/mTOR pathway, suppressing cleaved caspase-3 and PARP, and promoting paclitaxel resistance^28^. Similarly, in hepatocellular carcinoma, NEAT1-driven lipid metabolic remodeling sustains AKT/mTOR activity and enhances cell survival under therapeutic stress^29^, ^30^. Furthermore, NEAT1 is involved in multidrug resistance through upregulation of ABC transporters, and regulates cell proliferation by modulating cyclin-dependent kinases such as Cyclin D1^28^. NEAT1 is also known to be upregulated upon DNA damage^31^, ^32^. These findings highlight NEAT1 as a pleiotropic regulator of tumor cell survival under chemotherapy stress. While prior studies in ovarian cancer have shown that exosomal NEAT1 from M2-polarised macrophages promotes immune escape and drug resistance^33^, whether NEAT1 plays a similar role in DDP-induced TAMs in lung cancer remains unknown.

Previous studies have implicated NEAT1 in chemoresistance across multiple cancers and suggested its exosomal transfer under stress conditions^28^, ^33^. Furthermore, NEAT1 is known to be upregulated in response to DNA damage and to facilitate DNA repair via paraspeckle assembly^31^, ^32^. Given that DDP exerts cytotoxicity primarily through DNA damage, we reasoned that NEAT1 might be a key lncRNA enriched in exosomes from DDP-treated macrophages. Here, we hypothesise that DDP-induced macrophages upregulate NEAT1, package it into exosomes, and deliver it to A549 cells, where it enhances DNA damage response and reduces apoptosis. In particular, NEAT1 may exert its effects via transcriptional activation of MAD1L1 and suppression of p53 signalling. This study aims to dissect the EV-lncRNA-target gene axis in DDP-treated TAMs and elucidate its role in mediating DDP resistance in lung adenocarcinoma.

## Results

### Effect of DDP-treated macrophages on A549 cell viability and NEAT1 expression and changes in NEAT1 expression under DDP-treated stress

**Figure 1:**
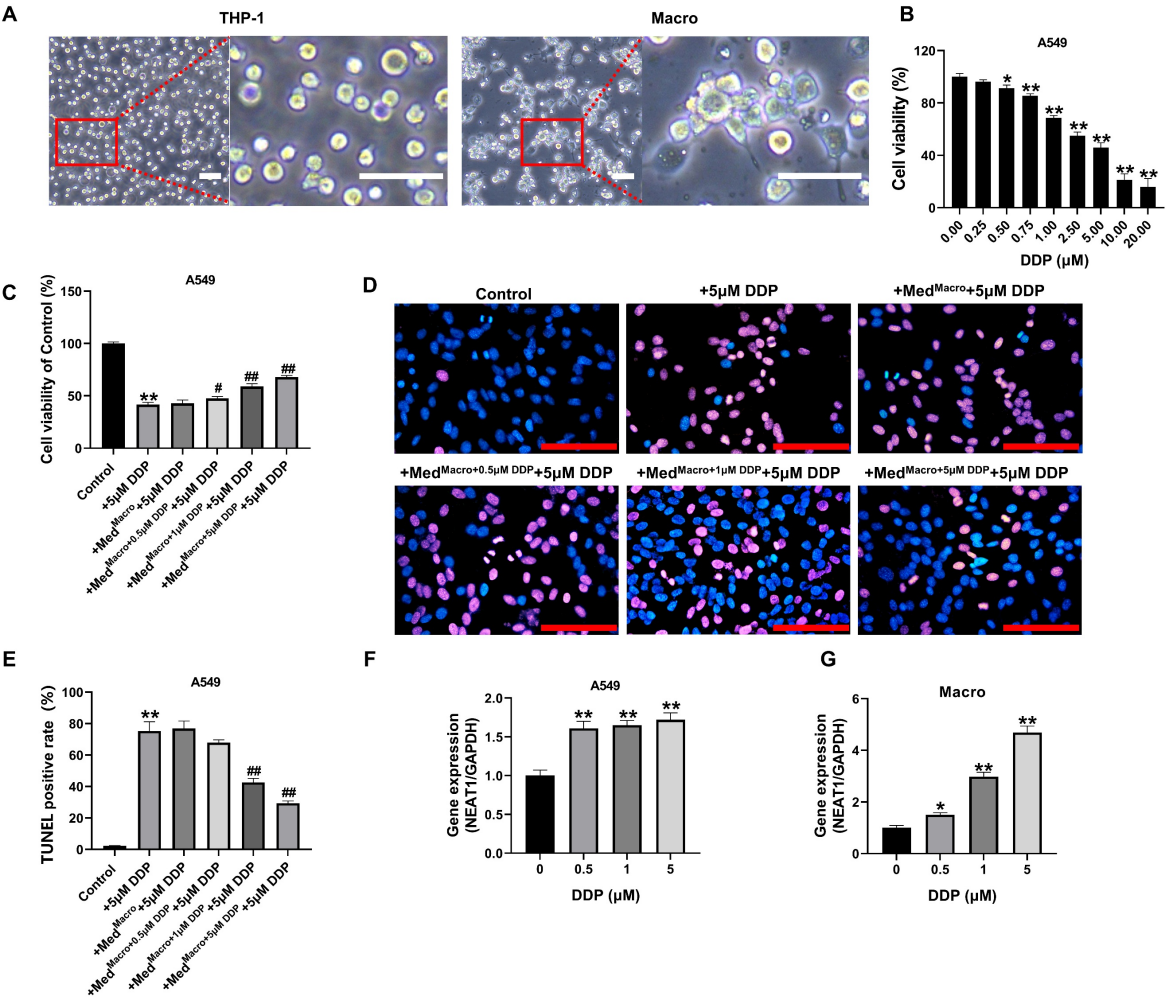
Effect of DDP-treated macrophages on A549 cell viability and apoptosis, and on NEAT1 expression in response to DDP. (A) Macrophage-like induction of THP-1 cells by PMA observed by phase contrast microscopy; (B) MTT assay for the effect of DDP on A549 cell activity; (C) MTT was used to detect the effect of different concentrations of DDP-treated macrophage culture supernatants on the activity of A549 cells under 5 μM pressure; (D-E) TUNEL assay was performed to quantify DNA fragmentation (apoptotic cells); (F-G) qRT-PCR was performed to detect the expression level of NEAT1.Scale bars= 100 μm. Data are presented as mean ± SD (n = 3 independent biological replicates). Statistical significance was determined by one-way ANOVA with Tukey's post hoc test. SD indicates error bars, *p < 0.05*, *p < 0.01, #p < 0.05 and ##p < 0.01. Med^Macro^, Med^Macro+0.5 μM DDP^, Med^Macro+1μM DDP^, Med^Macro+5 μM DDP^ indicate macrophage-conditioned medium treated with0, 0.5, 1, 5 μM DDP, respectively.

THP-1 cells were induced with 200 ng/ml PMA. As shown in Figure 1A, the cells were transformed from suspension to an adherent state and extended to pseudopods, indicating successful induction into macrophages. Lung cancer A549 cells were treated with different concentrations of DDP, and A549 cell proliferation was significantly inhibited at DDP concentrations > 0.5 μM (*P* < 0.05, Figure 1B). We subsequently assessed whether a DDP-treated macrophage-conditioned medium could alleviate the inhibitory effect of DDP on A549 cells. The results showed that macrophage-conditioned medium supplemented with different concentrations of DDP significantly increased the viability of A549 cells (*P* < 0.05, compared with that of the DDP-alone group, Figure 1C). This protective effect was DDP dose-dependent. TUNEL staining showed significantly fewer apoptotic cells in A549 cultures treated with macrophage-conditioned medium plus DDP (1-5 μM) versus DDP alone (*P* < 0.01, Figure 1D-E). In addition, we assessed NEAT1 expression in response to DDP stress. qRT-PCR results revealed that DDP treatment (0.5-5 μM) significantly upregulated NEAT1 expression in A549 cells compared to the untreated control, although this induction did not follow a strict dose-response pattern across concentrations (Figure 1F). In contrast, when macrophages were treated with DDP, NEAT1 expression exhibited a clear, gradient-increasing trend with rising DDP concentration, demonstrating significant concentration dependence (Figure 1G, P < 0.05 compared to the 0 μM DDP group).

### Exosome-mediated transfer of NEAT1 from DDP-treated macrophages activates DNA damage response

**Figure 2:**
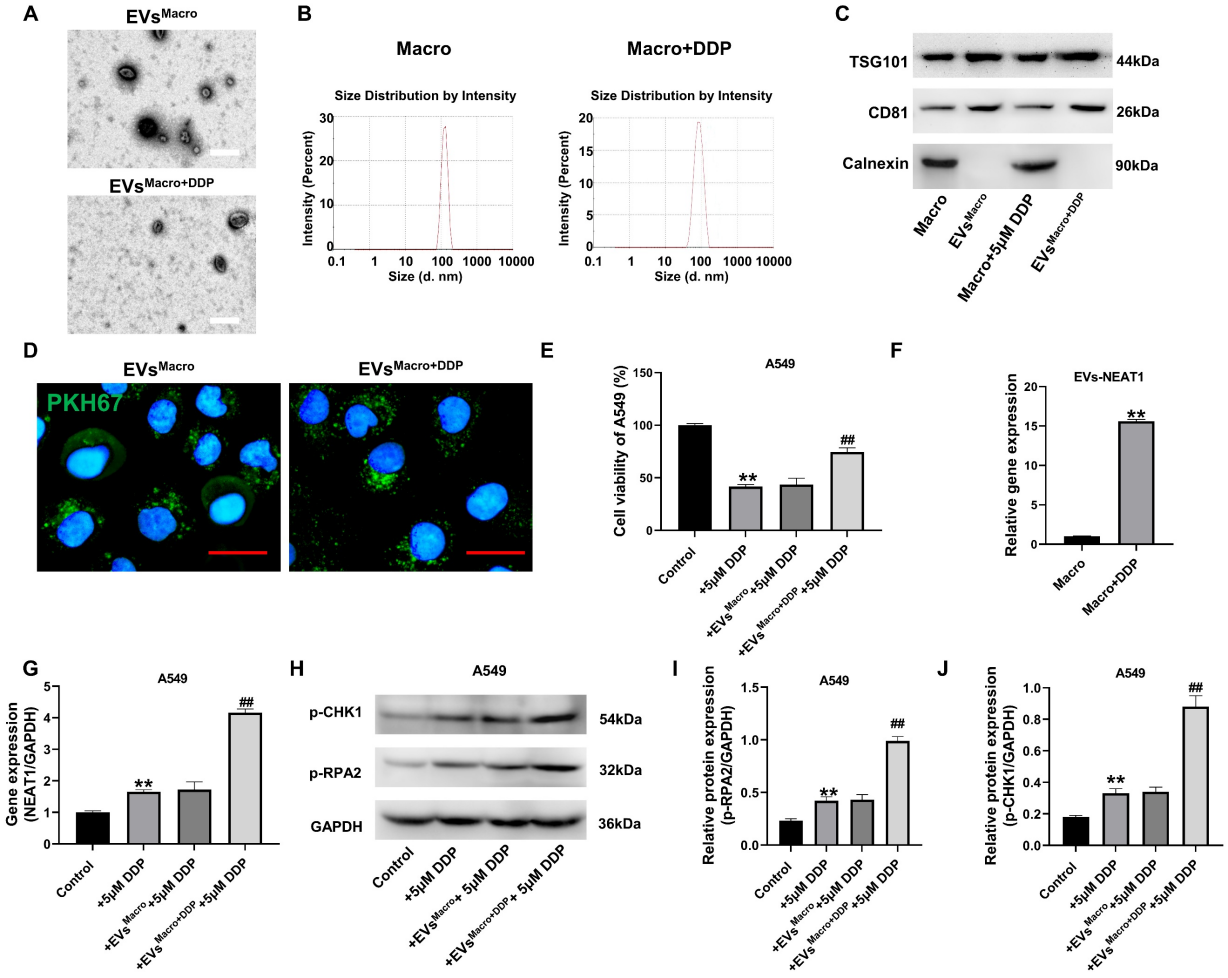
Exosomal transfer of NEAT1 from DDP-treated macrophages activates the DNA damage response in recipient A549 cells. (A) TEM was performed to observe the morphology of the exosomes. Scale bars= 200 nm. (B) NTA was used to determine the exosome particle size. (C) Western blot analysis of exosomal markers (TSG101, CD81) and negative control calnexin. (D) Uptake of the A549 cells after PKH67 labeling of the exosomes. Scale bars= 25 μm. (E) MTT assay for the effect of exosomes on the activity of DDP-treated A549 cells; (F-G) qRT-PCR was performed to detect NEAT1 expression levels; (H-J) Immunoblot analysis was performed to detect the phosphorylation levels of the RPA2 and CHK1 proteins. Data are presented as mean ± SD (n = 3 independent biological replicates). Two-tailed Student's t-test for (F) and one-way ANOVA with Tukey's post hoc test was performed for others. SD indicates error bars, *p < 0.01 and ##p < 0.01. EVs^Macro^, EVs^Macro+DDP^ indicate Extracellular vesicles originate from macrophages treated with 0, 5 μM DDP, respectively.

Based on the reported role of NEAT1 in DNA damage response and its presence in exosomes, we first isolated exosomes from untreated and DDP-treated macrophages and assessed their NEAT1 content. Exosomes were extracted and purified for identification. The results of electron microscopy showed that both types of exosomes had a double-layer molecular membrane structure, and all the exosomes were in the shape of a round bowl (Figure 2A); the size of the exosome particles was approximately 100-150 nm (Figure 2B); the exosome markers TSG101 and CD81 were detected in the isolated vesicles, while the endoplasmic reticulum protein Calnexin was absent, confirming the purity of our exosome preparations (Figure 2C). Additional positive markers (CD9 and CD63) are shown in Supplementary Figure S1A; the BCA assay revealed comparable exosomal protein concentrations across groups with DDP treatment or not (Figure S1B). Fluorescence microscopy confirmed PKH67-labeled exosome uptake by A549 cells. Exosomes from both types of macrophages were taken up by A549 cells (Figure 2D). Normal macrophage exosomes had no significant effect on DDP-treated cell activity damage, while DDP-treated macrophage exosomes significantly upregulated the cell activity level of A549 cells under DDP-treated stress (Figure 2E); macrophage exosomes treated with DDP significantly upregulated NEAT1 expression compared with that in the Macro group (*P <* 0.01); A549 cells given DDP-treated macrophage exosomes significantly upregulated NEAT1 expression compared with that in both the DDP group and the EVs^Macro^ group (*P* < 0.01, Figure 2F-G). Western blotting demonstrated (Figure 2H-J) that the levels of phosphorylated RPA2 (p-RPA2) and phosphorylated CHK1 (p-CHK1), markers of DNA damage response activation, were significantly increased after EVs^Macro+DDP^ treatment, and the differences were statistically significant (*P* < 0.01) compared with those in the EVs^Macro^ group.

### Effect of differential NEAT1 expression on DDP sensitivity and DNA damage response in A549 cells

**Figure 3:**
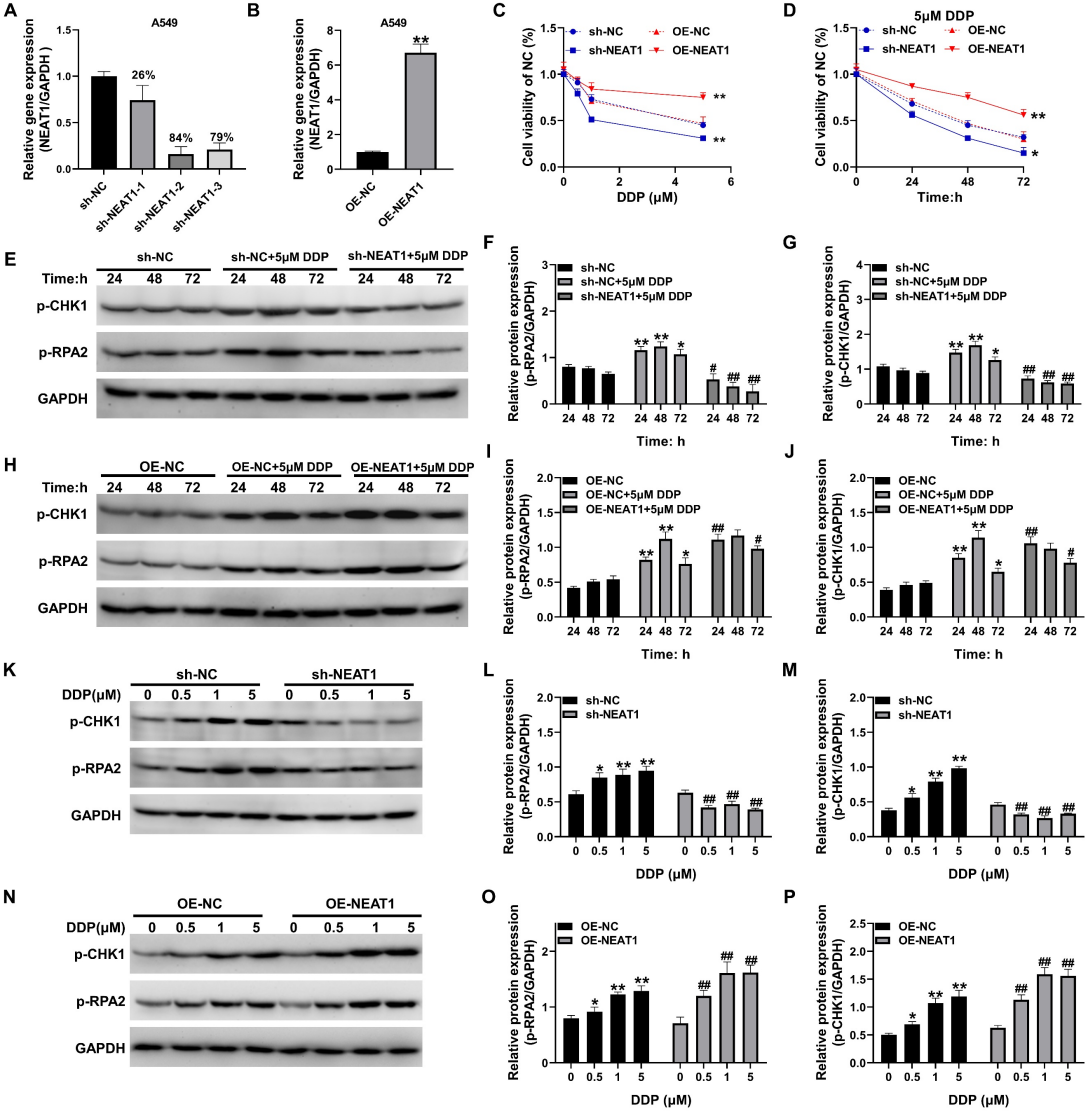
Effect of differential NEAT1 expression on DDP sensitivity and DNA damage repair mechanisms in A549 cells. (A-B) qRT-PCR was used to detect the efficiency of NEAT1 silencing and overexpression; (C-D) MTT was used to detect the change in activity in response to DDP treatment after silencing and overexpression of NEAT1; (E-P) Western blotting was used to detect the levels of p-CHK1 and p-RPA2 under different treatment conditions. Data are presented as mean ± SD (n = 3 independent biological replicates). Two-tailed Student's t-test for (B) and one-way ANOVA with Tukey's post hoc test was performed for others. SD indicates error bars, *P < 0.05, *P < 0.01, #*P* < 0.05 and ##*P* < 0.01. sh-NC, sh-NEAT1-1, sh-NEAT1-2, sh-NEAT1-3, OE-NC, OE-NEAT1 indicate A549 cell, NEAT1 silencing-A549 cell -1, NEAT1 silencing-A549 cell -2, NEAT1 silencing-A549 cell -3, A549 cell, NEAT1 overexpression-A549 cell, respectively.

The silencing and overexpression of the lncRNA NEAT1 in A549 cells were combined with a lentiviral assay. The qRT-PCR results showed that NEAT1 shRNA2 had the most significant effect on the silencing of NEAT1 in A549 cells, with a silencing efficiency of 84%, and the pCDH overexpression plasmid significantly upregulated the expression of NEAT1. Compared to the OE-NC group, the difference was statistically significant (*P* < 0.01, Figure 3A-B). MTT assays revealed that overexpression of NEAT1 significantly upregulated A549 cell activity in the presence of 1-5 μM DDP while silencing NEAT1 significantly downregulated A549 cell activity in the presence of 1-5 μM DDP (*P* < 0.05, Figure 3C-D). The levels of p-RPA2 and p-CHK1 were significantly increased in A549 cells after DDP treatment and started to decrease at 72 h, and the differences were statistically significant (*P* < 0.05, Figure 3E-G) compared with those in the sh-NC group. However, after silencing NEAT1, the expression levels of phosphorylated RPA2 and phosphorylated CHK1 proteins were significantly decreased, and the protein expression levels were significantly lower than those in the sh-NC group at 24-72 h (*P* < 0.05, Figure 3E-G). After the overexpression of NEAT1, the levels of both phosphorylated RPA2 and phosphorylated CHK1 proteins rapidly increased to a peak at 24 h, and they remained elevated from 24 to 72 h post-treatment, the levels of phosphorylated RPA2 and CHK1 in the OE-NEAT1+DDP group were significantly greater than those in the OE-NC+DDP group, and the differences were statistically significant (*P* < 0.05, Figure 3H-J). OE-NEAT1 not only rapidly upregulated the phosphorylation of RPA2 and CHK1 in response to DDP but also maintained these higher levels for a more extended period. Notably, NEAT1 overexpression sustained RPA2/CHK1 phosphorylation, silencing NEAT1 significantly inhibited the expression of the RPA2 and CHK1 proteins under DDP-treated stress, impairing the DNA damage response mediated by RPA2/CHK1 phosphorylation, whereas overexpression of NEAT1 significantly upregulated the phosphorylation of the RPA2 and CHK1 proteins, and the levels of responsive phosphorylated proteins increased as the concentration of DDP increased. These differences reached statistical significance versus respective controls at all time points (P < 0.05; Figure 3K-P).

### Effects of NEAT1 and macrophage-derived exosomes on the cell cycle and apoptosis of A549 cells

**Figure 4:**
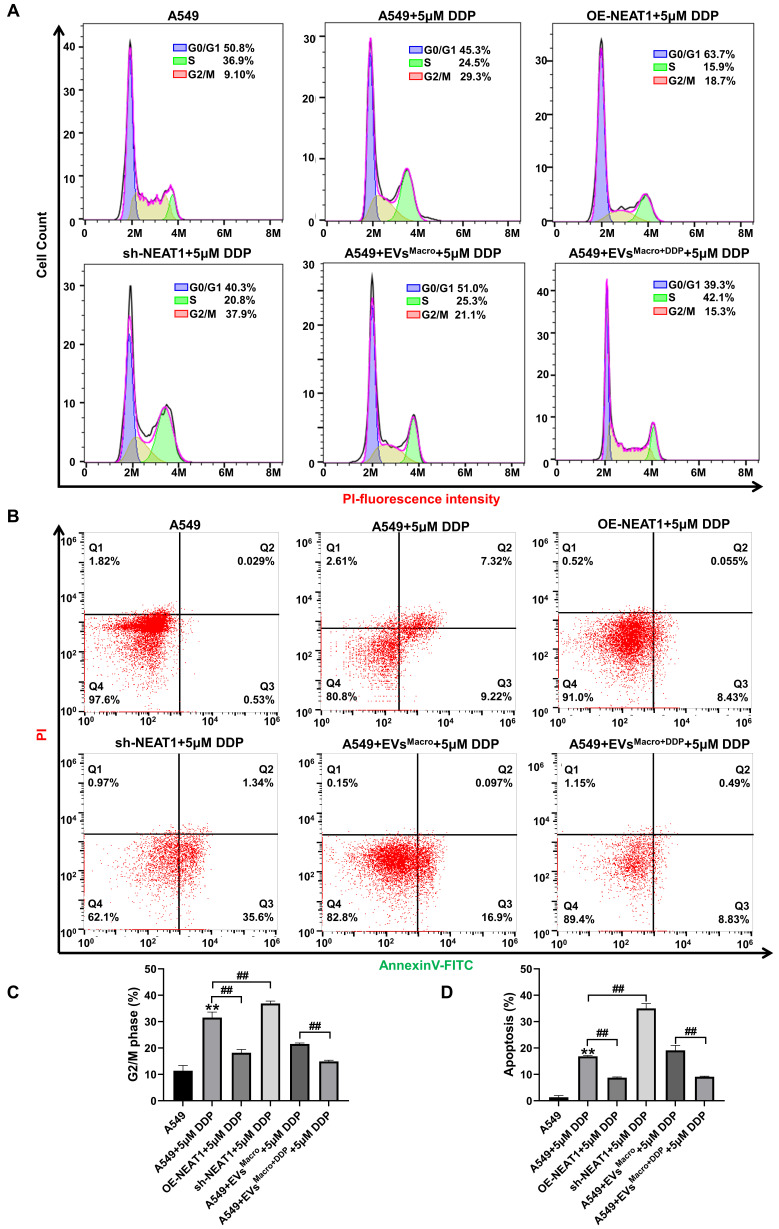
Effect of NEAT1 and macrophage-derived exosomes on the cell cycle and apoptosis of A549 cells. (A) Flow cytometry was used to detect cell cycle changes. (B) Flow cytometry was used to detect changes in apoptosis after Annexin V-FITC/PI double staining. (C) Data were analyzed using FlowJo software (v10.8.1) to quantify the proportion of cells in G2/M phase. (D) FlowJo V10 was used to quantify the proportion of cells in apoptosis. Data are presented as mean ± SD (n = 3 independent biological replicates). Statistical significance was determined by one-way ANOVA with Tukey's post hoc test. SD indicates error bars, **P < 0.01 and ##*P* < 0.01. OE-NEAT1, sh-NEAT1 indicate NEAT1 overexpression-A549 cell, NEAT1 silencing-A549 cell, respectively.

The G2/M phase of A549 cells was blocked after DDP treatment (*P* < 0.01, Figure 4A, C), and overexpression of NEAT1 promoted the progression of the G2/M phase to the next cell cycle. Silencing NEAT1, on the other hand, significantly enhanced the DDP-treated G2/M arrest (*P* < 0.01, Figure 4A, C); under DDP-treated stress, DDP-treated macrophage exosomes significantly reduced the percentage of A549 cells in the G2/M phase, and the difference was statistically significant compared with that in the EVs^Macro^ group (*P* < 0.01, Figure 4A, C). Flow cytometric analysis of Annexin V/PI staining showed NEAT1 overexpression significantly reduced the number of apoptotic cells, NEAT1 silencing significantly increased the level of DDP-treated apoptosis, and the difference was statistically significant in both groups compared with the DDP group (*P* < 0.01, Figure 4B, D). DDP-treated macrophage exosomes significantly reduced the percentage of apoptotic A549 cells under DDP-treated stress, and the difference was statistically significant compared with that in the EVs^Macro^ group (*P* < 0.01, Figure 4B, D). Together, the flow cytometry and TUNEL assay results established that NEAT1 and macrophage-derived exosomes confer DDP resistance by mitigating cell cycle arrest and apoptosis. Given that DDP exerts its cytotoxic effects primarily by causing DNA damage, we reasoned that the observed phenotypes likely stemmed from enhanced DNA damage response. Therefore, we next investigated the underlying molecular mechanism, focusing on the role of NEAT1 in the assembly of key protein complexes involved in the DNA damage response (See Figure S2). RNA immunoprecipitation (RIP) assays revealed that NEAT1 targets PRPF19 and CDC5L. Furthermore, co-immunoprecipitation (Co-IP) experiments showed that NEAT1 overexpression enhanced the assembly of the PRPF19/BCAS2/PLRG1/CDC5L complex, an effect that was potentiated by exosomes from DDP-treated macrophages (Figure S2A, B). Similarly, NEAT1 facilitated the formation of the NONO/SFPQ/FUS complex (Figure S2C, D). Importantly, RNA fluorescence in situ hybridization (FISH) combined with immunofluorescence demonstrated that DDP treatment induced the nuclear co-localization of NEAT1 transcripts with the paraspeckle protein PSPC1 (Figure S2E), providing spatial context for these interactions. Collectively, these results indicate that exosomal NEAT1 bolsters the DNA damage response by stabilizing critical nuclear protein complexes.

### Transcriptome analysis of NEAT1 involvement in downstream regulatory targets of DDP resistance in A549 cells

**Figure 5:**
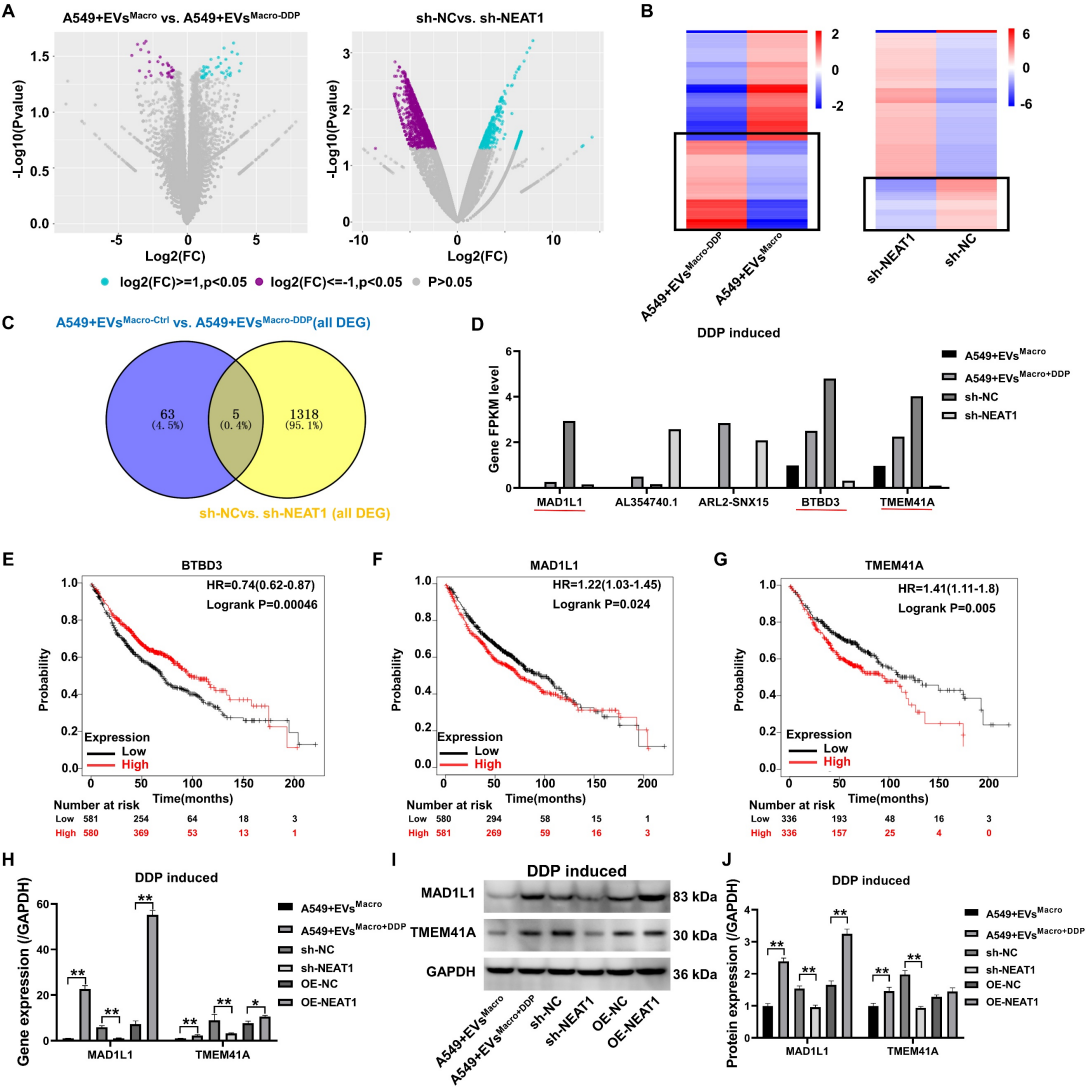
Transcriptome analysis of NEAT1 involvement in downstream regulatory targets of DDP resistance in A549 cells. (A) Volcano plot showing gene expression genes; (B) Heatmap analysis of differentially expressed genes; (C) Venn diagram analysis of common target genes significantly upregulated in EVs^Macro+DDP^ and downregulated after silencing NEAT1; (D) Bar plot showing the expression levels of the candidate target genes in transcriptome sequencing; (E-G) Kaplan-Meier survival analysis (https://kmplot.com) of target genes at the prognostic level in lung cancer; (H) qRT-PCR to detect the gene expression levels of MAD1L1 and TMEM41A; (I-J) Western blotting to detect the protein expression levels of MAD1L1 and TMEM41A. Data are presented as mean ± SD (n = 3 independent biological replicates). Statistical significance was determined by a one-way ANOVA with Tukey's post hoc test. SD indicates error bars, **P < 0.01.

Volcano plots of RNA-seq data revealed significant regulation of genetic signaling in A549 cells under EVs^Macro^ and sh-NEAT1 treatment conditions (Figure 5A). KEGG and Reactome analysis reveal that this treatment activated multiple oncogenic signaling pathways (Figure S3A-D). By heatmap analysis (Figure 5B), we focused on genes that were significantly upregulated in the EVs^Macro+DDP^ group and downregulated after NEAT1 was silenced. Venn diagram (Figure 5C) revealed five coregulated target genes: MAD1L1, lncRNA AL354740.1, ARL2-SNX15 readthrough transcript, BTBD3, and TMEM41A. The results of the expression levels of the target genes in the transcriptome showed (Figure 5D) that three genes were significantly upregulated in the EVs^Macro+DDP^ group and significantly downregulated after silencing NEAT1: MAD1L1, BTBD3, and TMEM41A. The prognostic values of the above three genes in lung cancer were analyzed through the Kaplan-Meier plotter online website (Figure 5E-G), and the results suggested that high expression of the MAD1L1 and TMEM41A genes was significantly associated with poorer lung cancer prognosis. The qRT-PCR results showed that both the EVs^Macro+DDP^ and OE-NEAT1 strains significantly upregulated the expression of the MAD1L1 and TMEM41A genes (Figure 5H). Analysis of the magnitude of regulation revealed that NEAT1 overexpression induced a 7.7-fold upregulation of MAD1L1 transcripts, whereas the TMEM41A gene was upregulated by 1.36-fold only after overexpression of NEAT1. Western blotting results showed that EVs^Macro+DDP^ and OE-NEAT1 significantly upregulated MAD1L1 protein expression (*P* < 0.01, Figure 5I-J), but OE-NEAT1 had no significant effect on TMEM41A protein abundance (*P* = 0.44, Figure 5I-J).

### NEAT1 targets the MAD1L1 promoter to regulate the cell cycle and apoptosis via the p53 pathway

**Figure 6:**
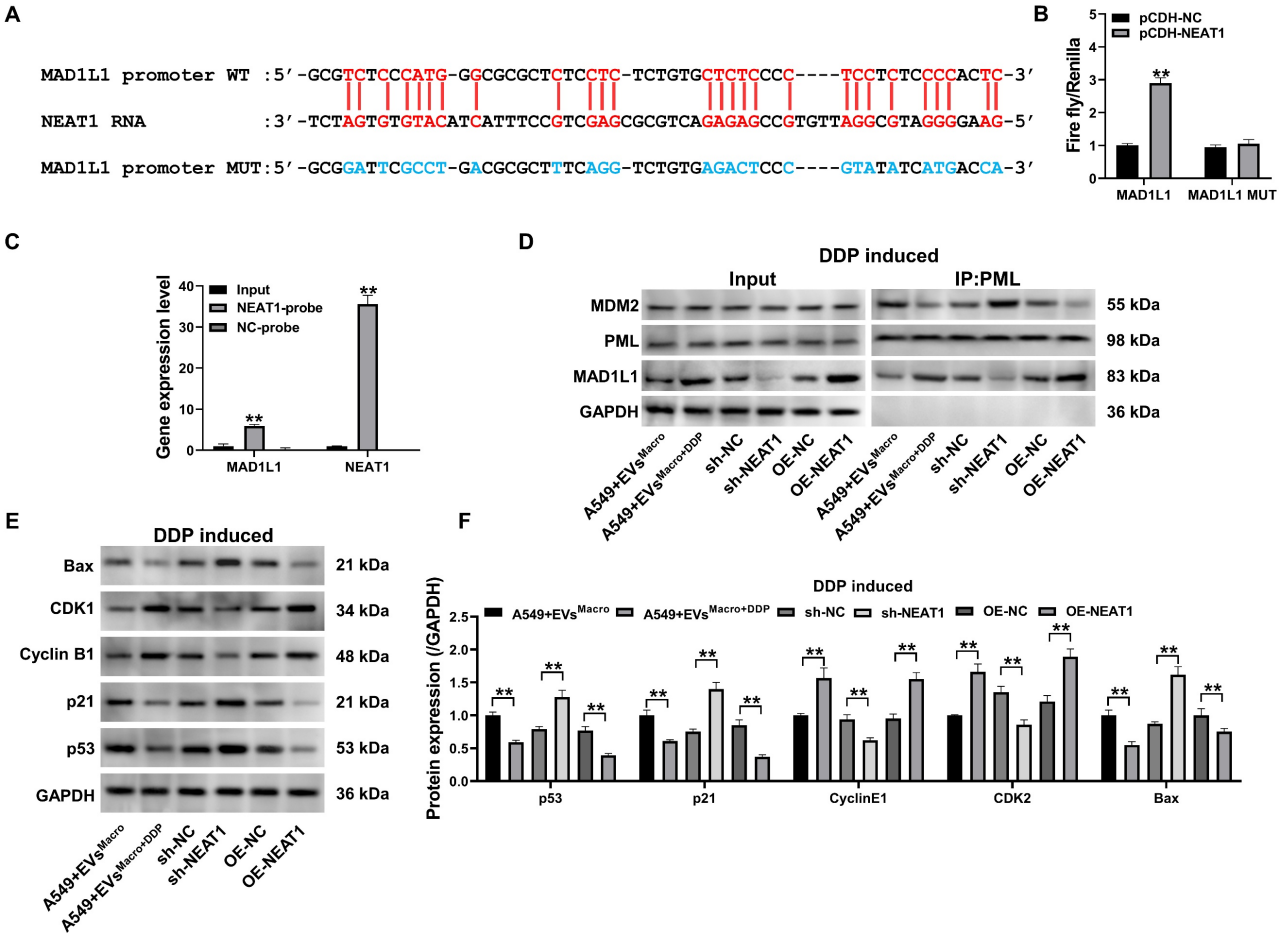
NEAT1 targeting the MAD1L1 promoter regulates the cell cycle and apoptosis through the p53 pathway. (A) NEAT1 RNA and MAD1L1 gene promoter sequence targeting the binding sequence site; (B) Dual luciferase activity reporter assay results; (C) qPCR analysis showing the enrichment of the MAD1L1 promoter region DNA precipitated by biotinylated NEAT1-specific probes; (D) Co-IP to detect the changes in the expression of the PML coprecipitated proteins MAD1L1 and MDM2; (E-F) Western blotting to detect changes in p53/p21/Cyclin B1/CDK1/Bax protein expression levels. Data are presented as mean ± SD (n = 3 independent biological replicates). Statistical significance was determined by a one-way ANOVA with Tukey's post hoc test, SD indicates error bars, **P < 0.01. DDP induced indicate cells treated with 5 μM DDP.

Combined with a sequence comparison of the NEAT1 RNA sequence with the MAD1L1 promoter region sequence, NEAT1 was determined to potentially target the MAD1L1 gene promoter sequence (Figure 6A). Overexpression of NEAT1 significantly upregulated the relative fluorescence intensity of the wild-type MAD1L1 group (*P* < 0.01, Figure 6B), as confirmed by a dual-luciferase reporter assay using MAD1L1 promoter constructs in which the MAD1L1 promoter-targeted binding sequence was mutated. This finding indicated that NEAT1 upregulated the transcription level of the plasmid containing the wild-type MAD1L1 promoter sequence, while overexpression of NEAT1 did not significantly change the mutant MAD1L1 promoter sequence (*P* > 0.05, Figure 6B). To determine whether NEAT1 targets the MAD1L1 promoter region, we performed an RNA-chromatin pull-down assay. Biotinylated NEAT1-specific probes were used to precipitate NEAT1 and its associated chromatin complexes from crosslinked cells. qPCR analysis of the precipitated DNA revealed a significant enrichment of the MAD1L1 promoter sequence, but not a control genomic region, in the NEAT1 pull-down sample compared to the input chromatin (P < 0.01, Figure 6C). This result demonstrates that NEAT1 is enriched at the MAD1L1 promoter, supporting its role in transcriptional regulation. Co-IP results (Figure 6D) revealed that EVs^Macro+DDP^ and OE-NEAT1 enhanced MAD1L1-PML protein interaction and decreased the coprecipitation of MDM proteins. In contrast, sh-NEAT1 inhibited the level of MAD1L1 precipitation with PML proteins but increased the intensity of MDM2 coprecipitation with PML proteins. Western blotting further confirmed that EVs^Macro+DDP^ and OE-NEAT1 significantly upregulated the protein expression of Cyclin B1 and CDK1 while downregulating the protein expression of p53/p21/Bax (*P* < 0.01, Figure 6E-F). In contrast, sh-NEAT1 significantly downregulated the protein expression of Cyclin B1 and CDK1 but upregulated the protein expression of p53/p21/Bax.

### Silencing MAD1L1 reverses the effects of NEAT1 overexpression on apoptosis and cell cycle progression in DDP-treated A549 cells

**Figure 7:**
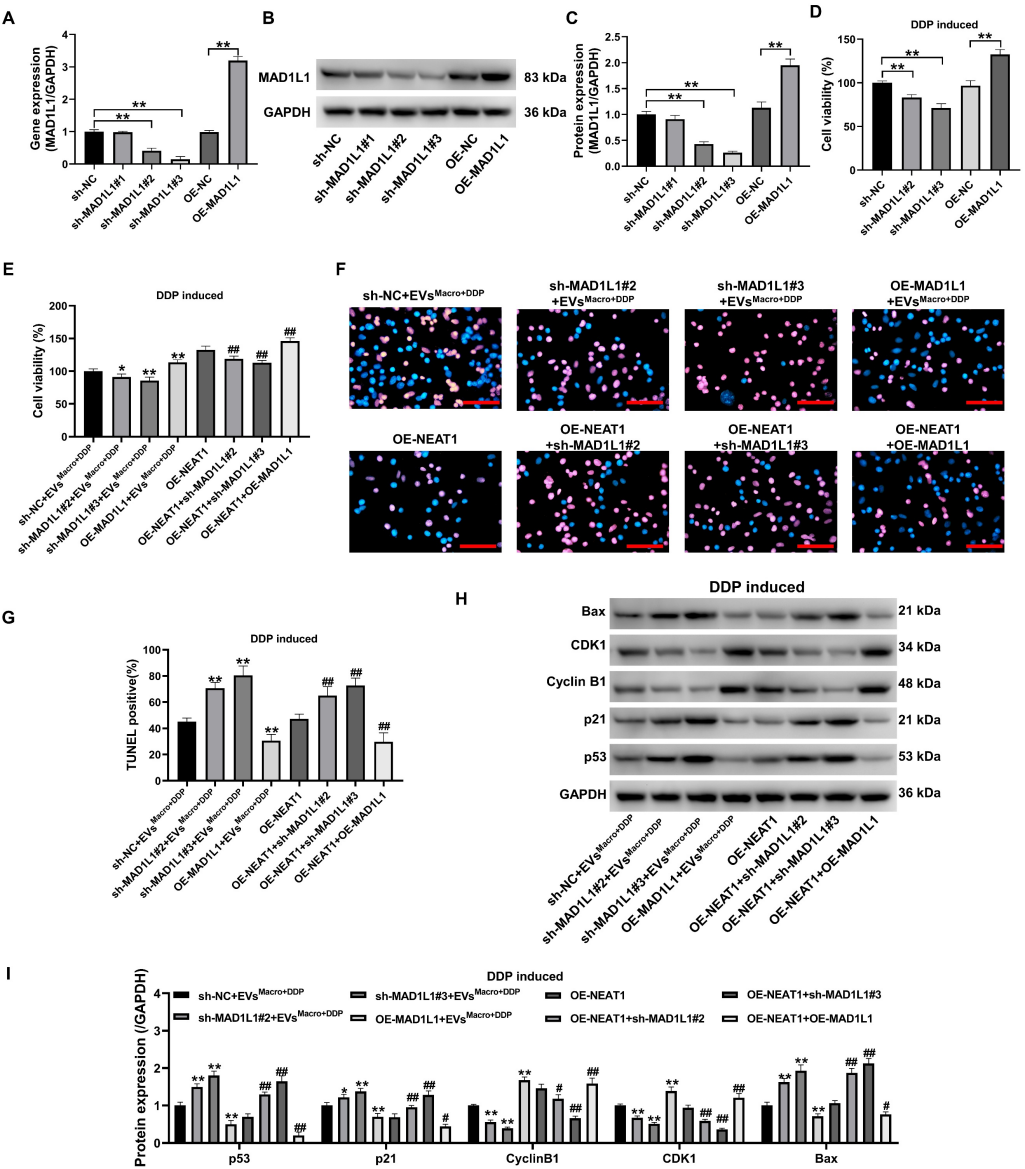
DDP-treated regulation of A549 cell apoptosis and cell cycle progression by MAD1L1 silencing. (A) qRT-PCR was used to detect the silencing and overexpression efficiency of the MAD1L1 gene in A549 cells; (B-C) Western blotting was used to detect the silencing and overexpression efficiency of the MAD1L1 protein; (D-E) MTT was used to detect changes in the cellular activity level; (F-G) Quantification of TUNEL-positive cells (apoptotic cells); (H-I) Western blotting was used to detect changes in the p53/p21/cyclin B1/CDK1/Bax protein expression levels. Scale bars= 100 μm. Data are presented as mean ± SD (n = 3 independent biological replicates). Statistical significance was determined by one-way ANOVA with Tukey's post hoc test. SD indicates error bars, *P < 0.05, **P < 0.01, #*P* < 0.05 and ##*P* < 0.01. sh-NC, sh-MAD1L1#1, sh-MAD1L1#2, sh-MAD1L1#3, OE-NC, OE-MAD1L1, OE-NEAT1 indicate A549 cell, MAD1L1 silencing-A549 cell #1. MAD1L1 silencing-A549 cell #2. MAD1L1 silencing-A549 cell #3, A549 cell, MAD1L1 overexpression-A549 cell, NEAT1 overexpression-A549 cell, respectively.

To verify that MAD1L1 is an important pathway through which NEAT1 participates in DDP resistance in A549 cells, this study first inhibited and overexpressed the MAD1L1 gene (Figure 7A) and protein (Figure 7B-C) in A549 cells. The cellular activity of A549 cells was significantly downregulated after MAD1L1 was silenced, while MAD1L1 overexpression significantly upregulated the cellular activity of A549 cells under DDP-treated stress (*P* < 0.01, Figure 7D). Silencing MAD1L1 significantly downregulated A549 cell activity under 5 μM DDP, and the difference was statistically significant compared with that in the EVs^Macro+DDP^ group and OE-NEAT1 group (*P* < 0.05, Figure 7D). Notably, MAD1L1 overexpression potentiated NEAT1's effects to upregulate A549 cell activity, and the difference was statistically significant compared with that in either the EVs^Macro+DDP^ group or OE-NEAT1 group (*P* < 0.05, Figure 7E). Silencing MAD1L1 significantly increased the number of TUNEL-positive cells (Figure 7F-G) and the p53/p21/Bax protein expression levels in A549 cells under 5 μM DDP-treated stress but decreased the Cyclin B1/CDK1 protein expression levels (Figure 7H-I); these findings were significantly different from those in the EVs^Macro+DDP^ group and OE-NEAT1 group (*P <* 0.05). Notably, MAD1L1 overexpression potentiated with overexpression of NEAT1 to decrease the percentage of TUNEL-positive cells and downregulate the p53/p21/Bax protein expression level in A549 cells. The overexpression of MAD1L1 synergized with the overexpression of NEAT1 to downregulate TUNEL positivity and p53/p21/Bax protein expression in A549 cells while upregulating Cyclin B1/CDK1 protein expression, with statistically significant differences compared to those in the EVs^Macro+DDP^ group or the OE-NEAT1 group (*P* < 0.01).

### Study of the effect of EVs^Macro+DDP^ and NEAT1 silencing on DDP treatment of lung cancer in nude mouse xenograft model

**Figure 8:**
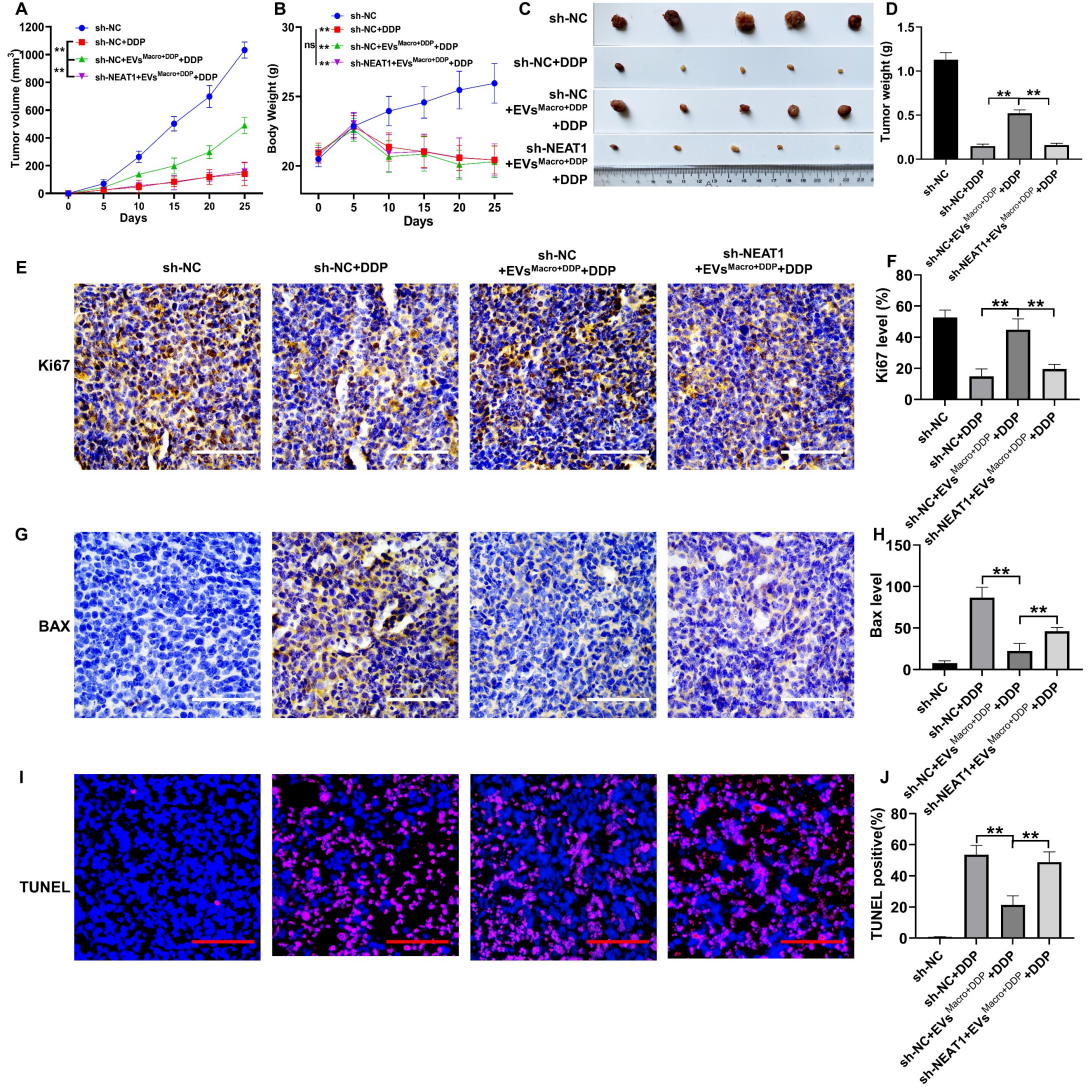
Study of the effect of EVs^Macro+DDP^ and NEAT1 silencing on DDP-treated lung cancer in BALB/c nude mice. (A) Tumor volume change trend graph during the treatment period; (B) Body weight change curve of nude mice during the treatment period; (C) Representative images of excised tumors at the study endpoint; (D) Quantitative analysis of excised tumor weights; (E-F) Positive rate of Ki67 staining detected by immunohistochemistry; (G-H) Immunohistochemistry to detect the intensity of Bax protein expression and quantification of immunohistochemical staining intensity (Image-Pro Plus 6.0) ; (I-J) TUNEL staining results and quantitative statistics of positive rate by IPP6.0 Statistics.Scale bars= 100 μm. Data are presented as mean ± SD (n = 5 independent biological replicates for panels A-D, and n = 3 independent biological replicates for panels E-J). Statistical significance was determined by a one-way ANOVA with Tukey's post hoc test. SD indicates error bars, **P < 0.01. sh-NC, sh-NC+DDP, sh-NC+EVs^Macro+DDP^+DDP, sh-NEAT1+EVs^Macro+DDP^+DDP indicate BALB/c nude mice were injected by A549 cells without treatment, BALB/c nude mice were injected by A549 cells and treated by DDP, BALB/c nude mice were injected by A549 cells and treated by DDP and EVs^Macro+DDP^, BALB/c nude mice were injected by sh-NEAT1-A549 cells and treated by DDP and EVs^Macro+DDP^, respectively.

The tumor volume and body weight of the mice were monitored throughout the study. DDP significantly inhibited the tumor volume of the xenografts compared to the sh-NC group (*P* < 0.01, Figure 8A). Conversely, EVs^Macro+DDP^ treatment significantly increased the tumor volume compared to the DDP group (*P* < 0.01, Figure 8A). This pro-tumor effect was significantly reversed by NEAT1 silencing (*P* < 0.01, Figure 8A). All DDP-treated groups showed a significant decrease in body weight compared to the sh-NC group (*P* < 0.01, Figure 8B). However, no significant differences in body weight were observed among the three DDP-treated groups, indicating that neither EVs^Macro+DDP^ nor sh-NEAT1 significantly exacerbated or alleviated the systemic toxicity associated with DDP treatment (Figure 8B). At the study endpoint, representative tumor images visually corroborated the findings on tumor volume and weight (Figure 8C). Consistent with the volume data, DDP significantly reduced the excised tumor weight, which was increased by EVs^Macro+DDP^ co-treatment (*P* < 0.01, Figure 8D). NEAT1 silencing again effectively counteracted the increase in tumor weight induced by EVs^Macro+DDP^ (*P* < 0.01, Figure 8D). Immunohistochemistry and TUNEL staining for pathological examination of the tumor tissues suggested that EVs^Macro+DDP^ significantly elevated the Ki-67 proliferation index of the tumors and decreased the intensity of Bax protein and the positivity rate of TUNEL staining, and the difference was statistically significant compared with that in the DDP group (*P* < 0.01, Figure 8E-J). Silencing NEAT1 in the A549 group significantly reversed the regulatory effect of EVs^Macro+DDP^ on the above phenomena in the tumors, and the difference between the EVs^Macro+DDP^ group and the sh-NEAT1-EVs^Macro+DDP^ group was statistically significant (*P* < 0.01). We further assessed whether the MAD1L1/p53 axis is modulated by macrophage-derived exosomes and NEAT1 in the xenograft tumors. qRT-PCR analysis confirmed that both NEAT1 and MAD1L1 transcript levels were significantly elevated in the EVs^Macro+DDP^ group compared to the DDP group (*P* < 0.01, Figure S4A-B). Silencing NEAT1 in A549 cells effectively counteracted this upregulation. At the protein level, western blotting of tumor lysates revealed that intervention with EVs^Macro+DDP^ significantly upregulated the expression of MAD1L1, Cyclin B1, and CDK1, while downregulating the expression of p53 and p21, compared to DDP treatment alone (*P* < 0.01, Figure S4C-H). Notably, the silencing of NEAT1 significantly reversed the regulatory effects of EVs^Macro+DDP^ on these proteins, bringing their levels closer to those observed in the DDP control group. These *in vivo* results solidify the conclusion that macrophage-derived exosomes deliver NEAT1 to cancer cells, where it activates the MAD1L1/p53 axis to promote cell cycle progression and suppress apoptosis, thereby driving DDP resistance.

## Discussion

In this study, guided by prior evidence linking NEAT1 to exosome-mediated chemoresistance and DNA repair, we identified NEAT1 as a key lncRNA enriched in exosomes from DDP-treated macrophages. We further demonstrate that these exosomes deliver NEAT1 to A549 lung adenocarcinoma cells, where it drives DDP resistance via a novel NEAT1-MAD1L1-p53 signaling pathway. This finding reveals a new mechanism of tumor microenvironment-mediated chemoresistance. Notably, macrophage-derived exosomal NEAT1 has been implicated in promoting cancer progression in other contexts-for example, NEAT1-enriched exosomes from inflammatory macrophages enhanced colorectal cancer growth and stemness^34^. Our work extends these observations to lung adenocarcinoma: we show that exosomal NEAT1 is transferred to A549 cells and upregulates MAD1L1, thereby blunting p53-dependent apoptosis and fostering DDP resistance. Thus, to our knowledge this is the first report linking macrophage-derived NEAT1 to MAD1L1-p53 signaling in the context of DDP resistance.

These findings build on the well-established roles of NEAT1 in nuclear paraspeckles and stress responses. NEAT1 is the architectural scaffold of paraspeckles -membrane-less nuclear bodies that are rapidly induced under stress to regulate gene expression and DNA damage response^35^, ^36^. Paraspeckles contain numerous RNA-binding proteins and are anchored on NEAT1: in particular, binding of NONO/SFPQ to NEAT1 is critical for paraspeckle assembly^37^, ^38^. Functionally, paraspeckles sequester proteins and RNAs to modulate pathways such as the DNA damage response; for instance, paraspeckles are induced within minutes after ionizing radiation, recruiting repair factors (like RPLP0) to sites of DNA damage and enhancing DNA-PK-mediated double-strand break repair^36^, ^39^. Importantly, p53 itself upregulates NEAT1 in response to DNA damage (forming a negative feedback loop), and NEAT1-driven paraspeckles are known to affect replication stress and chemotherapeutic sensitivity^31^, ^40^. In this context, our results add a new dimension: exosomal NEAT1 not only participates in paraspeckle architecture but also actively suppresses p53 signaling via MAD1L1. In other words, NEAT1 both senses genotoxic stress (through paraspeckles) and modulates the p53 checkpoint, creating a potent feed-forward mechanism that favors cell survival and contributes to chemoresistance under chemotherapy.

These insights complement and extend previous work on NEAT1 in chemoresistance. Elevated NEAT1 has been repeatedly linked to therapy resistance in multiple cancers. For example, NEAT1 overexpression in NSCLC cells was shown to induce paclitaxel resistance by enhancing caspase-3 expression and activating the Akt/mTOR pathway^41^. More broadly, NEAT1 promotes resistance by regulating apoptosis, the cell cycle, DNA repair, drug efflux, epithelial-mesenchymal transition, and stemness^42^. Our study specifically implicates MAD1L1 (a mitotic checkpoint protein)[43]as a new mediator of NEAT1's effects. This adds to the growing understanding of how lncRNAs orchestrate chemoresistance through diverse mechanisms, including the regulation of autophagy and mRNA stability^44^, ^45^. Aberrations involving MAD1L1 are known to confer resistance^46^, ^47^: notably, a RARS-MAD1L1 fusion was shown to induce stem-like properties and resistance to chemo- and radiotherapy in nasopharyngeal carcinoma^48^. Consistent with this, we find that NEAT1 upregulates MAD1L1 in NSCLC cells, suppressing p53-dependent checkpoints. By linking a tumor microenvironment lncRNA to a mitotic checkpoint protein, our work uncovers a previously unrecognized intersection of lncRNA and cell-cycle control in chemoresistance.

The clinical implications of these findings are significant. Exosomal lncRNAs are highly stable in blood^49^, ^50^ and are attractive biomarkers: circulating NEAT1 levels might predict or monitor DDP resistance and macrophage involvement in patients with lung adenocarcinoma. In fact, targeting NEAT1 in macrophage-derived exosomes markedly suppressed tumor growth in a CRC model^34^, suggesting that similar interventions in lung cancer could resensitize tumors to chemotherapy. Therapeutic strategies could include antisense oligonucleotides or CRISPR-based silencing of NEAT1 to restore p53 function, or small-molecule inhibitors of NEAT1-mediated interactions^28^. Additionally, modulating tumor-associated macrophages (for example, blocking M2 polarization or exosome release, or using CSF1R inhibitors) might prevent delivery of NEAT1 to cancer cells^51^. Thus, the NEAT1-MAD1L1-p53 axis offers multiple potential targets: NEAT1 itself, the MAD1L1 checkpoint pathway, and the macrophage-tumor communication. Exploiting these could provide novel approaches to overcome DDP resistance in NSCLC.

We acknowledge several limitations. First, while our *in vitro* and xenograft models provide mechanistic insights, the findings lack direct validation in clinical samples from NSCLC patients. Future studies should measure exosomal NEAT1 levels in patient plasma or tumor tissues to correlate them with DDP response and prognosis, which is essential for establishing its translational relevance. Second, most mechanistic data were obtained using cell lines and simplified co-culture systems; it will be important to confirm these pathways in more physiologically relevant models, such as patient-derived xenografts (PDX). Third, we used GAPDH as an internal control in stressed cells; however, GAPDH expression can vary under stress and in cancer, which may affect normalization^52^. Future studies should validate our findings using multiple reference genes or exogenous controls. Fourth, most data were obtained in cell lines and *in vitro* models; it will be important to confirm these mechanisms *in vivo*, for example using patient-derived xenografts (PDX) or genetically engineered mouse models. Finally, while we confirmed the uptake of macrophage-derived exosomes by A549 cells, the specific endocytic pathways and receptor interactions facilitating this process remain to be elucidated and represent an important direction for future research. Prospective clinical evaluation is also needed: if validated in patient cohorts, circulating exosomal NEAT1 could serve as a non-invasive biomarker for DDP resistance, and therapeutic strategies targeting the NEAT1-MAD1L1-p53 axis could be explored in early-phase trials. Future studies should also investigate potential crosstalk between NEAT1 and other resistance-associated pathways, such as those involving autophagy regulation^44^ or alternative polyadenylation events^45^, to develop more effective combination therapies. Such efforts will be crucial for translating our mechanistic discovery into clinical impact.

In summary, this work uncovers a macrophage-derived exosomal NEAT1 mechanism of DDP resistance in lung adenocarcinoma via a novel NEAT1-MAD1L1-p53 signaling axis. By bridging NEAT1's known paraspeckle functions to checkpoint regulation, we highlight a unique lncRNA-mediated route of drug resistance. These insights not only advance understanding of NEAT1 biology in cancer but also suggest new biomarkers and therapeutic targets to improve outcomes in lung cancer patients.

## Materials and Methods

### Animal

Male BALB/c nude mice, aged six weeks and weighing 21 ± 1 g at the start of the experiment, were housed in SPF facility. All procedures involving animals were conducted in compliance with the guidelines approved by the Institutional Review Board of Shandong Provincial Hospital Affiliated to Shandong First Medical University Committee [Approval No. A-2019-027]. In accordance with the Institutional Animal Care and Use Committee (IACUC) protocols, euthanasia was performed via CO₂ inhalation once tumors grew to 15 mm in diameter.

### Cell culture

Human monocyte THP-1 cells and human lung adenocarcinoma A549 cells were purchased from Zhejiang Ruyao Biotechnology Co., Ltd. THP-1 cells were cultured in RPMI-1640 medium supplemented with 10% fetal bovine serum. Macrophage induction was performed according to Wan *et al.*^53^. THP-1 cells were induced to form macrophages by treatment with a final concentration of 200 ng/ml phorbol ester (PMA) for 24 h. A549 cells were cultured in a Ham's F-12K medium containing 10% fetal bovine serum. The cells were incubated at 37 °C with 95% humidity in a constant temperature incubator containing 5% CO_2_.

### MTT experiment

A549 cells were seeded at a density of 5 × 10^3^ cells per well into 96-well plates with 200 μl of growth medium and incubated at 37 °C for 24 hours. Subsequently, the medium was replaced with a serum-free medium, and the cells were subjected to various treatments for 48 hours. After treatment, 10 μL of 5 mg/mL MTT solution (Dojindo, Japan) was added to each well, and the plates were further incubated at 37 °C for 4 hours. After dissolving formazan crystals in 150 μL DMSO, absorbance was measured at 570 nm using a CMax Plus spectrophotometer (Molecular Devices, USA).

### qRT-PCR assay

Total RNA was extracted using TRIzol reagent (Invitrogen, USA), and 2 μg of RNA was reverse-transcribed into cDNA using the 1st Strand cDNA Synthesis Kit with gDNA removal (Novoprotein, China). Quantitative PCR was performed using SYBR qPCR SuperMix Plus (Novoprotein, China) on a 7500 Fast Real-Time PCR System (Applied Biosystems, USA). Relative gene expression levels were calculated using the 2^-ΔΔCt method. GAPDH was used as the internal control for normalization of lncRNA expression. The following primer sequences were used: NEAT1-Forward: 5'- GCT GGA CCT TTC ATG TAA CGG G-3', NEAT1-Reverse: 5'- TGA ACT CTG CCG GTA CAG GGA A-3'; MAD1L1-Forward: 5'-GTT GAA GGT CGA GGA GCT GGA A-3', MAD1L1-Reverse: 5'- GTT CAG GCT CAT GTG CAG CAC T-3'; GAPDH-Forward: 5'-GTC TCC TCT GAC TTC AAC AGC G-3', GAPDH-Reverse: 5'- ACC ACC CTG TTG CTG TAG CCA A-3'.

### NEAT1 overexpression and silencing cell construction

The NEAT1 overexpression plasmid pCDH-CMV-Neat1-copGFP-Puro was purchased from Zhejiang Ruyao Biotechnology Co. The NEAT1 silencing plasmid was constructed based on the classical sequence of NEAT1 in the NCBI database (ID: NR_028272), and three shRNA sequences targeting NEAT1 (NCBI ID: NR_028272) were designed using the GPP Web Portal online website. shRNA oligonucleotide sequences against NEAT1. The shRNAs were inserted into the pLKO.1 plasmid. The recombinant shRNA plasmid, in combination with the helper plasmid, was transfected into 293T cells using X-tremeGENE HP DNA Transfection Reagent (Roche). After 72 h, the cell cultures were collected, and the cellular debris was removed with disposable filters. A549 cells were incubated with a culture medium containing 2 µg/ml polybrene. After 48 h of infection, the cells were screened for resistance to a medium containing 2 µg/ml puromycin and maintained for 7-9 days. Finally, NEAT1 silencing and overexpression efficiency were measured by qRT-PCR.

### Exosome isolation

Exosomes for all functional and analytical experiments presented in this study were isolated from cell culture supernatant using differential ultracentrifugation, a widely established method for obtaining vesicles of high purity. Briefly, conditioned medium from macrophages was collected and subjected to sequential centrifugation steps to remove cells and debris: 300 ×g for 10 min, 2,000 ×g for 10 min, and 10,000 ×g for 30 min at 4°C. The clarified supernatant was then ultracentrifuged at 100,000 ×g for 70 min at 4 °C (Type 70 Ti rotor, Beckman Coulter) to pellet exosomes. The pellet was washed once with a large volume of phosphate-buffered saline (PBS) and subjected to a final ultracentrifugation step under the same conditions (100,000 ×g, 70 min). The final exosome pellet was resuspended in an appropriate volume of PBS for downstream applications. Protein concentration of the exosome preparation was determined using a bicinchoninic acid (BCA) assay.

### Exosome characterization protein assays

Exosomes from macrophages were collected and lysed by adding RIPA protein lysis buffer to obtain proteins. Western blotting was performed to detect the expression of the exosome-characterizing proteins TSG101 and CD81.

### Morphological and quantitative analysis of exosomes

To observe the morphology and ultrastructure of exosomes, the isolated vesicles were resuspended in 1 mL of PBS, and 3 μL was applied onto a copper mesh coated with a carbon-supported membrane. After allowing adsorption for 30 seconds, the grids were negatively stained with 2% uranyl acetate. Samples were then visualized under a Tecnai T120 transmission electron microscope, and images were captured with an FEI Eagle 4K × 4K CCD camera at 67,000 × magnification. For particle size distribution and concentration analysis, a NanoSight Malvern 3000 nanoparticle tracking analysis (NTA) system was used. This system enables direct measurement of particle size (30-1000 nm range) and particle concentration (particles/mL) in suspension based on Brownian motion. In addition, total exosomal protein content was measured using a BCA protein assay kit.

### Exosome uptake experiments

Extracted exosomes were labeled with the lipophilic fluorescent dye PKH67 (D0031, Solarbio) according to the manufacturer's instructions. To remove excess, unincorporated dye, the labeled exosome suspension was subjected to ultracentrifugation (100,000 ×g, 70 min) and washed once with a large volume of phosphate-buffered saline (PBS). The final pellet was resuspended in PBS. The protein concentration of the labeled exosome preparation was determined using a bicinchoninic acid (BCA) assay. A549 cells were seeded and grown to 70% confluence. Cells were then treated with PKH67-labeled exosomes (10 μg/ml, based on exosomal protein concentration) for 24 hours at 37 °C. As a critical control, a parallel group of cells was incubated with an equivalent volume of PKH67 dye solution that had been processed through the same ultracentrifugation and washing steps in the absence of exosomes (dye-only control). After incubation, cells were washed three times with PBS to remove any non-internalized exosomes or dye. The uptake of exosomes was then observed and recorded using a Leica DM500 fluorescence microscope.

### Western blotting

An appropriate amount of exosomal extract or cells was collected, washed with PBS, and lysed on ice using RIPA protein lysis buffer containing phosphatase inhibitors. Subsequently, the lysate was centrifuged and the supernatant was collected. Protein concentration was determined using BCA assay (Beyotime, China) with BSA standards. Protein samples (40 μg) were separated by SDS-PAGE, followed by transfer to PVDF membranes. The membranes were blocked for 2 h at room temperature using 5% skim milk. Subsequently, primary antibodies against TSG101 antibody(Abcam, UK, ab125011); CD81 antibody (Abcam, UK, ab109201); Calnexin antibody (Abcam, UK, ab22595);p-CHK1 antibody (ab79758, Abcam, UK);p-RPA2 antibody (ab109394, Abcam, UK); GAPDH antibody (A19056, ABclonal, China); MAD1L1 antibody (ab184560, Abcam, UK); TMEM41A antibody (ab236850, Abcam, UK);MDM2 antibody (4H26L4, Invitrogen, USA); PML antibody (ab72137, Abcam, UK); Bax antibody (ab32503, Abcam, UK); CDK1 antibody (ab133327, Abcam, UK, ); p21 antibody (ab109520, Abcam, UK); p53 antibody (ab32049, Abcam, UK); Cyclin B1 antibody (ab181593, Abcam, UK); CD9 antibody (A19027, ABclonal, China); CD63 antibody (ab216130, Abcam, UK); were incubated in 5% BSA/TBST at 4 °C for 12 h. On the following day, Goat anti-rabbit IgG-HRP-labeled secondary antibody (HA1001, Huabio, China) was added at a dilution of 1:2000 and incubated for 2 hours at room temperature. ECL chemiluminescent substrate was added for signal luminescence, and protein bands were detected using a ChemiDoc-It imaging system (UVP, USA). GAPDH was used as an internal reference protein for normalization, and the results were quantitatively and statistically analyzed by ImageJ software.

### Flow cytometry assays

To assess the cell cycle and apoptosis rates, the cells were first adjusted to a concentration of 5 × 10^5^ cells/mL, after which the suspensions were prepared. For cell cycle analysis, cells were fixed using 70% ethanol for 1 h at 4°C before being resuspended in PI/RNase staining solution containing 50 μg/mL propidium iodide and 100 μg/mL RNase A (A10798, Invitrogen, USA) and incubated for 15 min under light-avoidance conditions. For apoptosis analysis, cells were labeled using the Annexin V-FITC/PI Apoptosis Detection Kit (BMS500FI, Invitrogen, USA). Finally, combined cell cycle and apoptosis levels were detected using flow cytometry (Attune NxT, Thermo Fisher).

### RNA FISH and immunofluorescence assay

Following Emmanuelle *et al.*^54^, fluorescence in situ hybridization (FISH) of NEAT1 RNA was performed using a FISH assay kit (C10910, RiboBio, China) and a specific NEAT1 FISH probe (lnc1000059, RiboBio, China). First, the cells were fixed with 4% paraformaldehyde and then treated with a prehybridization solution of 1× PBS and 0.5% Triton X-100. This was followed by overnight hybridization with a fluorescently labeled NEAT1 probe in a hybridization buffer at 37 °C. Simultaneously, immunofluorescent labeling was performed using anti-PSPC1 antibody (1:200, D225417, Sangon) and an Alexa Fluor 488-coupled secondary antibody (4412S, Cell Signaling, USA). The cell nuclei were stained with DAPI for 5 min. Finally, images were acquired by fluorescence microscopy (DM500, Leica, Germany).

### Immunoprecipitation

Proteins were extracted from cells using RIPA lysis buffer, and Precleared protein lysates were incubated with antibody-conjugated protein A/G beads overnight: anti-PRPF19 antibody (MA5-32222 Invitrogen, USA), NONO antibody (A300-587A, Invitrogen, USA) and PML antibody (A301-167A, Invitrogen, USA). Then, the protein lysate was added. Then, 160 μL of protein lysate and 40 μL of loading buffer were added, and the mixture was heated at 100 °C for 5 min. The enriched proteins were then subjected to WB analysis with primary antibodies against BCAS2 (ab108330, Abcam, UK), PLRG (Cat no: ab86050, Abcam, UK), CDC5L (ab314000, Abcam), SFPQ (ab177149, Abcam, UK), and FUS (ab243880, Abcam, UK) diluted with Abcam at a dilution ratio of 1:1000.

### RNA-chromatin pull-down assay

The targeting of NEAT1 to the MAD1L1 promoter was assessed using an RNA-chromatin pull-down assay adapted from established protocols. Briefly, A549 cells (approximately 1 × 10^7^) were crosslinked with 1% formaldehyde for 10 min at room temperature. Glycine was added to quench the crosslinking. Cells were lysed, and chromatin was sheared by sonication to an average fragment size of 200-500 bp. Biotinylated DNA oligonucleotide probes complementary to NEAT1 (NEAT1 probe set) and negative control probes (LacZ probe set) were synthesized (RiboBio, China). Sheared chromatin was incubated with the probes overnight at 37 °C. Probe-bound complexes were captured using Dynabeads MyOne Streptavidin T1 (65601, Invitrogen, USA), followed by extensive washing. After reverse crosslinking and proteinase K digestion, DNA was purified. The enrichment of the MAD1L1 promoter region in the pulled-down DNA was quantified by qPCR using specific primers and normalized to the input chromatin DNA. Primers targeting its core promoter region (-184 to -58 bp relative to the transcription start site)^55^: Forward: 5'-CGG CCC TCC CAG CCA ATG C-3'; Reverse: 5'-TTT CTC CAG GCC AGG CGA GC-3'.

### RIP experiment

Cellular proteins were extracted from RIPA lysis buffer. Protein A/G magnetic beads coconjugated to the target antibody were added for incubation. After the target proteins bound to the protein A/G magnetic bead-antibody (PRPF19/CDC5L/NONO) complex were collected, the above magnetic bead-antibody complex was digested with 0.5 mg/mL proteinase K in 100 mM Tris-HCl (pH 7.5)/10 mM EDTA/0.5% SDS at 55 °C for 30 min. RNA precipitated from the protein complex was extracted by the TRIzol method, and the NEAT1 expression level was detected via qRT-PCR.

### Transcriptome analysis

Normal macrophage exosomes and DDP treated macrophage exosomes (100 μg/ml) were added to A549 cells, which were then treated with 5 μM DDP^17^, and RNA was extracted by the TRIzol method after incubation for 48 h. RNA was extracted from A549 cells in the sh-NEAT1 and sh-NC groups at the same time. The Illumina HiSeq sequencing platform was used for 50 bp single-end sequencing of the samples. The differentially expressed mRNAs were analyzed in each sample. The prognostic levels of target genes in lung cancer patients were analyzed via Kaplan-Meier Plotter (https://kmplot.com/analysis/).

### Dual luciferase activity reporter

The MAD1L1 promoter mutation sequence was constructed based on the binding sequence of NEAT1 targeted to the promoter region of the MAD1L1 gene. The dual luciferase reporter gene plasmid was constructed using the pGL3-Basic plasmid, and Renilla luciferase plasmid pRL-SV40 (E2231, Promega) was co-transfected at 1:10 ratio for normalization. The promoter region of MAD1L1 is a 2000 bp fragment before the start codon, and Zhejiang Ruyao Biologicals was commissioned to construct the wild-type and mutant MAD1L1 recombinant plasmids.

### MAD1L1 overexpression and silencing plasmid construction

MAD1L1 overexpression and silencing plasmids were purchased from Zhejiang Ruyao Biotechnology Co. The pCDH-CMV-Puro plasmid was used for overexpression plasmid construction, and the silencing plasmid used was pLKO.1. Cell transfection: A suitable number of cells were inoculated in 24-well plates to achieve a cell density of 50%-60% at the time of transfection, and the MAD1L1 shRNA1-3 (5 μg) or pCDH-MAD1L1 plasmid was transfected into A549 cells using LipofectamineTM 2000. MAD1L1 shRNA1-3 (5 μg) or the pCDH-MAD1L1 plasmid was transfected into A549 cells using LipofectamineTM 2000, and MAD1L1 gene and protein expression levels were detected after 48 h.

### TUNEL staining

A549 cells were treated according to the following subgroups: 1, the sh-NC+EVs^Macro+DDP^ group; 2, the sh-MAD1L1#2+EVs^Macro+DDP^ group; 3, the sh-MAD1L1#3+EVs^Macro+DDP^ group; 4, the OE-MAD1L1+EVs^Macro+DDP^ group; 5, the OE-NEAT1 group; 6, the OE-NEAT1+sh-MAD1L1#2 group; 7, the OE-NEAT1+sh-MAD1L1#3 group; and 8, the OE-NEAT1+OE-MAD1L1 group. Cells in groups 1-8 were cultured under 5 μM DDP treatment for 48 h. After 48 h, the DNA damage level was determined by using a TUNEL staining kit (C10618, Invitrogen, USA) to detect DNA fragmentation, a hallmark of apoptotic cell death. Images were observed and captured by fluorescence microscopy at 594 nm. The results were analyzed by Image-Pro Plus 6.0 software.

### Xenograft tumor model

Wild-type and sh-NEAT1-infected A549 cells (5 × 10^6^) were injected subcutaneously into the right axillary region of 6-week-old male BALB/c nude mice weighing 21 ± 1 g. Animals were grouped into the following groups: 1, sh-NC group; 2, sh-NC+DDP group; 3, sh-NC+EVs^Macro+DDP^+DDP group; and 4, sh-NEAT1+EVs^Macro+DDP^+DDP group. Tumor volumes were measured every 5 days after inoculation.5 days after cell injection, the DDP groups were injected with 4 mg/kg DDP via tail vein (three times per week), the EVs^Macro+DDP^ groups received peritumoral injections of 10 μg EVs (in 50 μL PBS) three times per week, concurrent with the DDP administration. The subcutaneous tumor and the sh-NC group were treated with PBS via matched administration routes. Twenty-five days after inoculation, the nude mice were euthanized by CO_2_ asphyxiation. Tumor tissues were fixed in 4% paraformaldehyde and then paraffin-embedded. The tumor tissues were immunoblotted to detect the protein expression levels of MAD1L1/p53/p21/Cyclin B1/CDK1, and qRT-PCR was performed to detect the gene expression levels of NEAT1/MAD1L1.

### Pathological testing

The deparaffinized tissue sections were placed in 0.01 M citrate buffer (pH = 6.0) and subjected to antigen retrieval in an autoclave at 121 °C for 20 min. Then, the sections were incubated with 3% H2O2 for 20 min. Then, they were incubated with 1% BSA at room temperature for 20 min, and Ki67 antibody (ab15580, Abcam, UK) and Bax antibody (ab32503, Abcam, UK) were added and incubated at 4 °C for 16 h. The primary antibodies were removed, and a drop of goat-anti-rabbit IgG-HRP secondary antibody (HA1001, Huabio, China) was added and incubated at room temperature for 1 h. DAB (A690009, Sangon, China) was used for color development. The nuclei were stained with hematoxylin. The cells were observed under a light microscope (DM500, Leica, Germany). Image-Pro Plus 6.0 software was used to quantify the IHC results. The level of DNA damage in the tumor tissues was assessed with reference to the Invitrogen TUNEL staining kit.

### Statistical analyses

All data are presented as the mean ± standard deviation (SD). For *in vitro* experiments, the value of “n” refers to the number of independent biological replicates. Unless otherwise specified, data from *in vitro* studies are derived from three (n = 3) independent biological replicates, and for *in vivo* xenograft studies, five (n = 5) mice were used per group. Comparisons between two groups were performed using an unpaired, two-tailed Student's t-test. A p value < 0.05 was considered statistically significant. All statistical analyses and graphing were performed using GraphPad Prism software (version 8.0).

## Supplementary Material

Supplementary figures.

## Data Availability

The datasets generated and/or analysed during the current study are available from the corresponding author on reasonable request.
