# Supplementary material for: Exosomal transfer of macrophage-derived NEAT1 enhances DNA damage response and confers cisplatin resistance in lung adenocarcinoma via the MAD1L1/p53 axis
Source: Int J Biol Sci. 2026 Mar 25;22(7):3807–25. doi: 10.7150/ijbs.128214 (PMC13086090; doi:10.7150/ijbs.128214)
Supplement: Supplementary file 1 — Supplementary figures. [file ijbsv22p3807s1.pdf]

Supplementary Figures

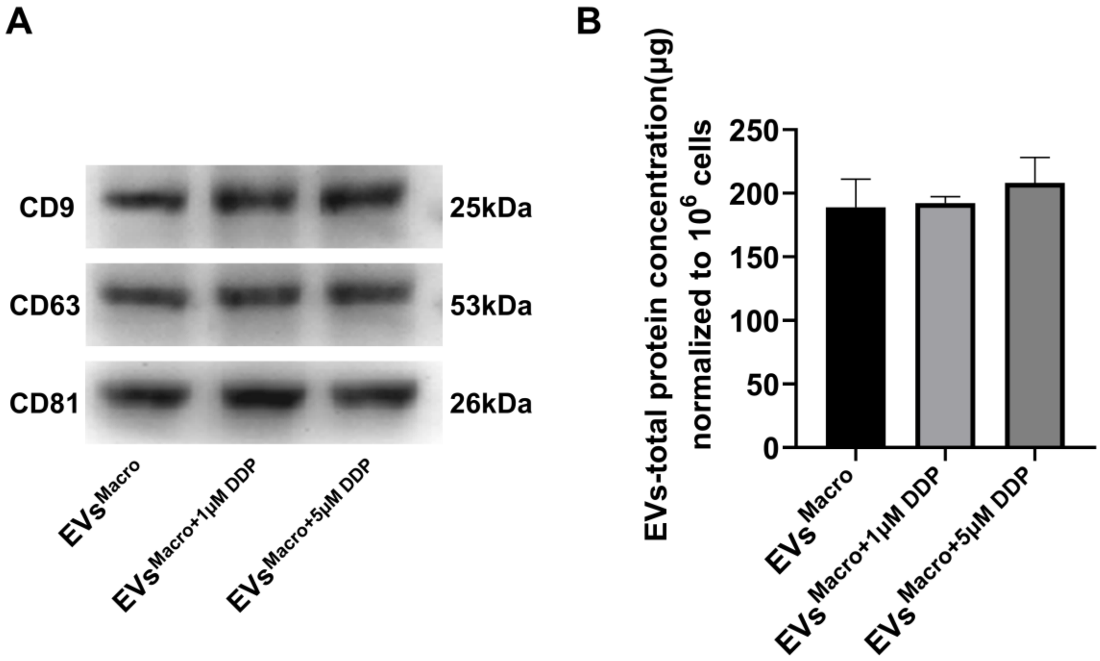

Figure S1. Identification of macrophage exosomes and the effects of DDP on macrophage-derived exosome secretion. (A) Measurement of the protein level of CD9, CD63, and CD81 of macrophage-derived exosomes by Western blotting. (B)The protein concentration of macrophage-derived exosomes was detected by the BCA method. Data are presented as mean ± SD (n = 3 independent biological replicates). Statistical significance was determined by one-way ANOVA with Tukey's post hoc test. SD indicates error bars, ns (not significant, P > 0.05) .EVs^{Macro}、$EVs^{Macro+1μM\ DDP}$ 、$EVs^{Macro+5\ μM\ DDP}$ indicate Extracellular vesicles originate from macrophages treated with0, 1, and 5 μM DDP, respectively.

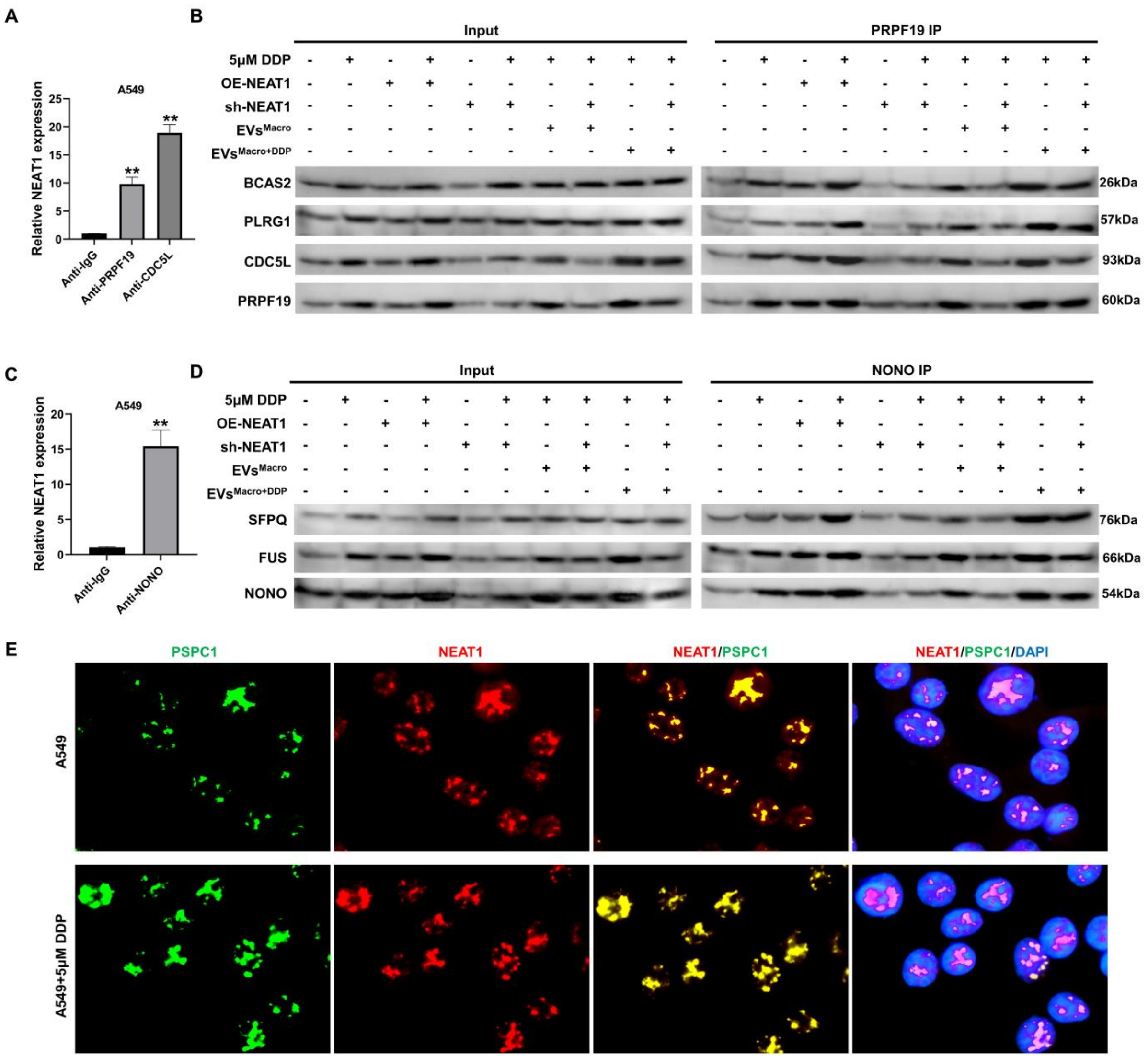

Figure S2. Effect of NEAT1 on DNA damage repair-associated protein complexes in A549 cells. (A) RIP assay to detect the expression level of NEAT1 after coprecipitation with PRPF19 or CDC5L proteins; (B) Co-IP assay to detect changes in protein expression of the proteins BCAS2/PLRG1/CDC5L coprecipitated with PRPF19 proteins; (C) RIP assay to detect the expression level of NEAT1 after coprecipitation with the NONO proteins; (D) Co-IP assay to detect changes in protein expression of SFPQ, FUS and NONO coimmunoprecipitated with NONO; (E) Immunofluorescence combined with FISH assay for colocalization analysis of

NEAT1 transcripts with PSPC1 protein.Scale bars= 25 μm. Data are presented as mean ± SD (n = 3 independent biological replicates). Two-tailed Student's t-test for (A) and one-way ANOVA with Tukey's post hoc test was performed for (C). SD indicates error bars, $^{**}P < 0.01$.

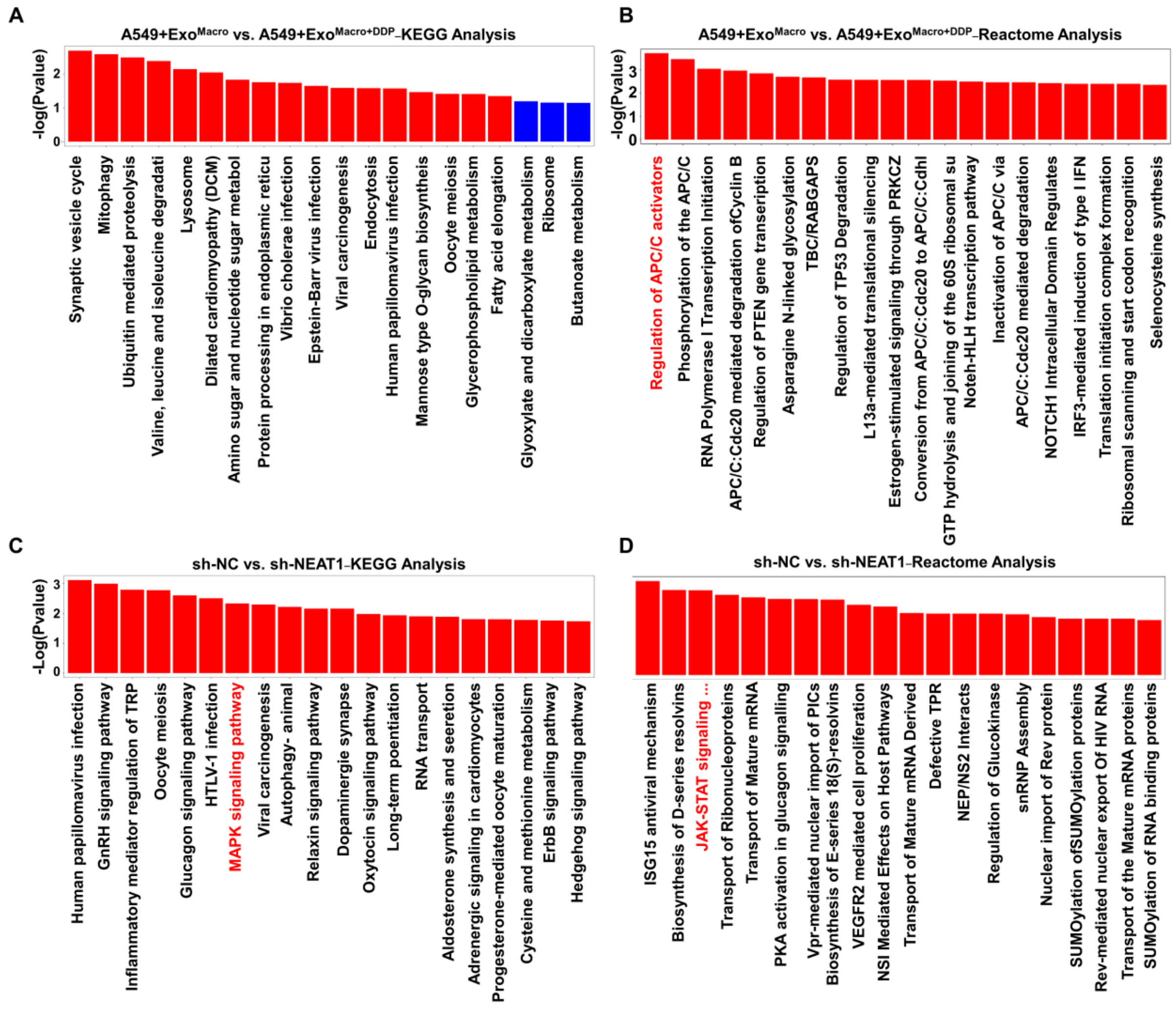

Figure S3. KEGG and Reactome analysis of NEAT1 involvement in downstream regulatory targets of DDP resistance in A549 cells. (A-B) KEGG (https://www.kegg.jp) and Reactome (https://reactome.org) pathway analysis for A549+Exo^{Macro} vs. A549+Exo$^{Macro+DDP}$. (C-D) KEGG and Reactome Analysis for sh-NC vs. sh-NEAT1.EVs^{Macro}, EVs$^{Macro+DDP}$ indicate Extracellular vesicles originate from macrophages treated with 0, 5 μM DDP, respectively.sh-NC, sh-NEAT1, indicate A549 cell, NEAT1 silencing-A549 cell, respectively.

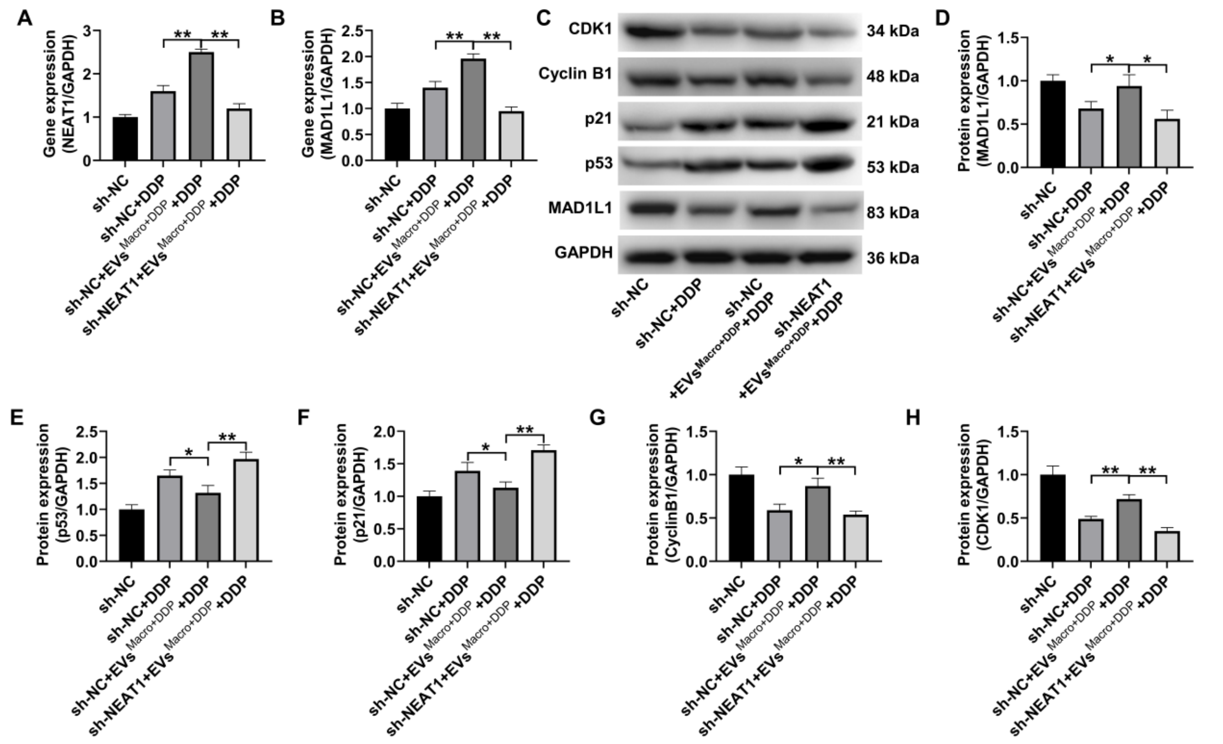

Figure S4. The MAD1L1/p53 pathway mediates the effects of EVs$^{Macro+DDP}$ and NEAT1 silencing on apoptosis and cell cycle-related proteins in nude mice.(A-B) qRT-PCR was used to detect the NEAT1 and MAD1L1 expression levels change in DDP or EVs treated nude mice' tumor tissues; (C-H) Western blotting was used to detect changes in the p53/p21/cyclin B1/CDK1/MAD1L1 protein expression levels. Scale bars= 100 μm. Data are presented as mean ± SD (n = 3 independent biological replicates). Statistical significance was determined by one-way ANOVA with Tukey's post hoc test. SD indicates error bars,*$P < 0.05$ and **$P < 0.01$.
